# Sentinel Lymph Node Detection Using SPECT and Gamma Probe in Low‐Risk Endometrial Cancer: Efficacy and Factors Associated With Detection Failure

**DOI:** 10.1111/ases.70015

**Published:** 2025-01-05

**Authors:** Kazuo Asanoma, Hideaki Yahata, Keisuke Kodama, Kaoru Okugawa, Masafumi Yasunaga, Ichiro Onoyama, Hiroshi Yagi, Shoji Maenohara, Kazuhisa Hachisuga, Takuro Isoda, Mototsugu Shimokawa, Kousei Ishigami, Yoshinao Oda, Kiyoko Kato

**Affiliations:** ^1^ Department of Obstetrics and Gynecology Kyushu University Hospital Fukuoka Fukuoka Japan; ^2^ Department of Clinical Radiology Kyushu University Hospital Fukuoka Fukuoka Japan; ^3^ Department of Biostatistics Yamaguchi University Graduate School of Medicine Ube Yamaguchi Japan; ^4^ Department of Anatomic Pathology Pathological Science, Graduate School of Medical Science, Kyushu University Fukuoka Fukuoka Japan

**Keywords:** endometrial neoplasm, minimally invasive surgical procedures, sentinel lymph node

## Abstract

**Introduction:**

This study examined factors that affected sentinel lymph node (SLN) identification of patients with endometrial cancer having a preoperative estimation of low recurrent risk.

**Methods:**

This study included 97 patients with endometrial cancer who attempted to identify SLN using a uterine cervical injection of technetium‐99 m phytate under laparoscopic or robotic‐assisted surgery at our institute. A preoperative single photon emission computed tomography (SPECT) and intraoperative gamma probe were used to detect hot nodes. Multiple clinical factors, including age, body mass index (BMI), and so on, were investigated for their association with SLN mapping failure.

**Results:**

Among 97 cases, SPECT failed to detect SLN unilaterally in 38 cases (39%) and on both sides in 9 cases (9%). Meanwhile, the gamma probe failed to detect SLN unilaterally in 23 cases (24%) and on both sides in 3 cases (3%). While only age was significantly associated with SLN detection failure using the SPECT detection system, both age and BMI were significantly associated with SLN detection failure using the gamma probe detection system. When limiting to the preoperative SLN detection failure cohort of 47 cases, there was a strong association between intraoperative SLN detection failure and BMI, but not age.

**Conclusion:**

The SLN biopsy system was effectively applied to patients with endometrial cancer who underwent minimally invasive surgery (MIS). Attempts to improve SLN identification in older patients and those with obesity are warranted to obtain maximum benefits of MIS for low‐ or medium‐risk cases.

## 1 Introduction

Endometrial cancer remains prevalent in advanced countries, including the United States, with an incidence rate of 8.0 per 100 000 women per year based on 2017–2021 data (National Cancer Institute: https://seer.cancer.gov/statfacts/html/corp.html). In Japan, the incidence rate of newly diagnosed endometrial cancer cases is 27.6 per 100 000 women per year based on 2019 data (National Cancer Center, https://ganjoho.jp/reg_stat/statistics/stat/cancer/18_corpus_uteri.html#anchor1). Fortunately, most cases are diagnosed at early stages, which are optimal indications for minimally invasive surgery (MIS), including laparoscopic surgery or robotic‐assisted surgery. While the operation time is longer, MIS is preferred in the appropriate cases due to a lower incidence of surgical site infection, transfusion, a shorter hospital stay, and earlier resumption of normal activities without adverse effects in perioperative complication and oncologic outcomes [1, 2, 3, 4, 5, 6, 7].

The optimal injection site of the tracer to detect sentinel lymph node (SLN) remains controversial, but the National Comprehensive Cancer Network (NCCN) guideline recommends cervical injection because of the easiness and reproducibility of the procedure [8, 9]. Meta‐analyses revealed that the tracer for the best detection rate of SLN may be a combination of indocyanine green and technetium‐99 m (^99m^Tc) radiolabeled colloid, and ^99m^Tc isotope alone detected SLN with enough high probability [10, 11]. Especially, the advantage of using a ^99m^Tc isotope is to detect deep signals penetrated through the fat tissue of patients with obesity [11].

Unsuccessful SLN detection occurs with a certain probability. Several studies reported that body mass index (BMI) [12, 13, 14, 15], age [12, 16], nonendometrioid histology [12, 13, 17], lymphovascular invasion [12, 17], and lymph node metastasis [13] were associated with SLN detection failure. There are several studies reporting detection rates of both single photon emission computed tomography (SPECT) and intraoperative gamma probe detection systems [18, 19]. However, there are no studies reporting detailed detection sites of the two modalities and clinical factors associated with SLN detection failure for each modality.

This study investigated the SLN detection efficiency of patients with endometrial cancer having a preoperative estimation of low recurrent risk who underwent MIS at our hospital. We used both SPECT and gamma probe detection systems and studied SLN detection rates, detection sites, and clinical factors associated with SLN detection failure for each modality.

## 2 Materials and Methods

### 2.1 Design and Setting

This retrospective cohort study recruited women with endometrial endometrioid carcinoma Grade 1 or 2 at estimated Stage IA who underwent MIS at Kyushu University Hospital from June 2019 to March 2023. Of the cases, 43 and 54 were treated laparoscopically and robotically, respectively. The Da Vinci Si system (Intuitive Surgical Inc., Sunnyvale, CA, USA) was used from June 2019 to July 2019 (in 2 cases), and the da Vinci Xi system from September 2019 to March 2023 (in 52 cases). Surgical modalities were randomly selected based on the availability of robotic instruments.

All patients with endometrial cancer considered for MIS underwent histological sampling of endometrial lesions, pelvic magnetic resonance imaging (MRI), and computed tomography. Preoperative clinical staging was identified based on the 2008 International Federation of Gynecology and Obstetrics (FIGO) criteria. A histological diagnosis, including grading, was preoperatively obtained. All patients provided informed consent preoperatively.

The laparoscopic surgeries involved eight gynecologic oncologists authorized by the Japan Society of Gynecologic Oncology. Meanwhile, robotic surgeries involved six gynecologic oncology surgeons authorized by the Japan Society of Gynecologic Oncology and certificated by Intuitive Surgical G.K., Japan.

Patients were placed in the lithotomy or supine position with side split, combined with the Trendelenburg position (10°–15° for laparoscopic surgery and 20°–25° for robotic surgery). Laparoscopic surgery involved five laparoscopic trocar installations in a diamond position, including a 5‐mm assistant trocar placed right to the umbilical position. Robotic surgery involved four da Vinci trocar installations in a horizontal line, including in the umbilical position. A 12‐mm assistant trocar was placed on the left side of the abdominal wall. General anesthesia with endotracheal intubation was applied to perform all surgeries. All cases underwent a simple hysterectomy and bilateral salpingo‐oophorectomy with SLN sampling. Lymphadenectomy, including the external iliac and obturator nodes, was performed on the side with unsuccessful SLN identification. When nodal metastasis was diagnosed by the intraoperative pathological examination, we converted the surgery to laparotomy, including a simple hysterectomy, bilateral salpingo‐oophorectomy, and omentectomy with whole pelvic and paraaortic lymph node dissection up to the renal vein height. Uteri that were too large to be vaginally extracted were collected via a mini‐laparotomy incision.

Surgical uterine and lymph node specimens were postoperatively applied for histological examination. Adjuvant therapy was proposed for patients with intermediate or high risk of recurrence assessed using predominantly used criteria [20, 21].

The Kyushu University institutional ethical committee provided ethical approval (approval number: 23109‐00).

### 2.2 SLN Detection

One day preoperatively, ^99m^Tc labeled sodium phytate colloid containing 148 MBq (40 mCi) per patient was injected into the subepithelium of the uterine cervix at four spots. Lymphoscintigraphy and SPECT images were obtained 2 h after the injection to locate the hot lymph nodes. A handheld gamma probe (neo2000, Neoprobe Corporation, Dublin, OH, USA) was used for intraoperative SLN detection. A lymph node having at least more than 10 times gamma counts above the background was recognized as an SLN. Intraoperatively, fat tissue was promptly removed from the lymph nodes by surgeons, and the isolated lymph nodes were submitted for intraoperative pathological examination.

### 2.3 Intraoperative Pathological Examination of SLN (Ultrastaging)

Intraoperatively, dissected SLNs were snap‐frozen and evaluated using 2‐mm‐interval slices along the short axis. Frozen sections from every slice stained with hematoxylin and eosin were diagnosed by institutional pathologists. In addition, SLNs were reevaluated after formalin fixation postoperatively.

### 2.4 Data Collection

Information on age, BMI (calculated as weight divided by the square of height), surgical modality, date of surgery, uterine tumor thickness measured using MRI, CA125, final histological grading, FIGO staging, myometrial invasion, lymphovascular invasion, lymph node metastasis, and pelvic cavity washing cytology were collected from the medical records at Kyushu University Hospital.

### 2.5 Statistical Analysis

Univariate and multivariate logistic analyses were conducted to extract clinical factors associated with SLN detection failure. Age, BMI, surgical modality, period when surgery was performed, uterine tumor thickness measured using MRI, CA125, final histological grading, FIGO staging, myometrial invasion, lymphovascular invasion, lymph node metastasis, and pelvic cavity washing cytology were analyzed for their association with SLN detection failure. Categorical variables were analyzed using Pearson's chi‐square test or Fisher's exact test for univariate analysis.

Before conducting multivariate analysis, factors belonging to a similar clinical category were excluded to avoid confounding among the clinical factors. A backward elimination method was used in multivariate logistic regression analysis to extract remarkable clinical factors associated with SLN detection failure. The exact odds ratios (ORs) were reported along with the 95% confidence intervals (CIs) and corresponding *p* values. We first performed univariate and multivariate analyses on all the 97 cases. We also performed analyses on 47 cases, which is a specific cohort of SLN detection failure using a preoperative SPECT.

JMP software (version 16; SAS Institute Inc., Cary, NC, USA) was used for all statistical analyses. A *p* < 0.05 was considered statistically significant.

## 3 Results

### 3.1 Patient Characteristic and SLN Detection Failure

A total of 97 patients with endometrial cancer underwent laparoscopic or robotic‐assisted hysterectomy were attempted to identify SLN preoperatively using SPECT imaging and intraoperatively using a gamma probe during the study period. Table 1 shows the characteristics of the cohort. Among 97 cases, preoperative SPECT failed to detect SLN unilaterally in 38 cases (39%) and on both sides in 9 cases (9%) (Table 1). Meanwhile, the intraoperative gamma probe failed to detect SLN unilaterally in 23 cases (24%) and on both sides in 3 cases (3%) (Table 1). We did not encounter any cases with empty packets. Two cases were observed to be Grade 3 and clear cell carcinoma. Fourteen cases were at more than Stage IB, including 3, 3, 1, 5, and 2 at Stages IB, II, IIIA, IIIC1, and IIIC2, respectively (Table 1).

**TABLE 1:** Patient demographic, clinical characteristics, and SLN detection failure.

		SPECT	SPECT	Gamma probe	Gamma Probe
Group	Total	Detection success	Detection failure	Detection success	Detection failure
Patients, *n*	97	47 (52%)	50 (48%)	71 (73%)	26 (27%)
Median age, years (range)	54.0 (22–80)	50.0 (22–73)	59.0 (32–80)	52.0 (22–80)	60.0 (32–80)
Median BMI (range)	25.0 (13.7–47.6)	23.8 (13.7–47.6)	26.3 (15.3–46.4)	23.8 (13.7–44.6)	28.9 (18.6–47.6)
Surgical modality, *n* (%)					
Laparoscopic	43 (44%)	19 (40%)	24 (48%)	32 (45%)	11 (42%)
Robotic‐assisted	54 (56%)	28 (60%)	26 (52%)	39 (55%)	15 (58%)
Number of surgeries each year, *n*					
2019	12	8	4	8	4
2020	25	12	13	19	6
2021	30	10	20	21	9
2022	24	12	12	18	6
2023	6	5	1	5	1
Thickness of tumor measured with MRI, mm (range)	13 (0–43)	13 (3–39)	12 (0–43)	12 (0–43)	14 (0–39)
Serum CA125, U/mL (range)	16.4 (5.5–103)	17.4 (7.1–49)	15.7 (5.5–103)	17.1 (5.5–103)	13.9 (8.2–66.8)
Histological grade, *n* (%)					
1 and 2	95 (98%)	47 (100%)	48 (96%)	69 (97%)	26 (100%)
3 and clear cell carcinoma	2 (2%)	0	2 (4%)	2 (3%)	0
FIGO stage, *n* (%)					
IA	83 (86%)	40 (85%)	43 (86%)	61 (86%)	22 (85%)
More than IB	14 (14%)	7 (15%)	7 (14%)	10 (14%)	4 (15%)
IB	3 (3%)	1 (2%)	2 (4%)	0	3 (11%)
II	3 (3%)	1 (2%)	2 (4%)	2 (3%)	1 (4%)
IIIA	1 (1%)	1 (2%)	0	1 (1%)	0
IIIC1	5 (5%)	3 (6%)	2 (4%)	5 (7%)	0
IIIC2	2 (2%)	1 (2%)	1 (2%)	2 (3%)	0
Myometrial invasion, *n* (%)					
Absent	40 (41%)	23 (49%)	17 (34%)	30 (43%)	10 (38%)
Present	57 (59%)	24 (51%)	33 (66%)	41 (57%)	16 (62%)
Myometrial invasion depth, *n* (%)					
< 1/2	89 (92%)	44 (94%)	45 (90%)	66 (93%)	23 (88%)
≥ 1/2	8 (8%)	3 (6%)	5 (10%)	5 (7%)	3 (12%)
Lymphovascular invasion, *n* (%)					
Absent	84 (87%)	42 (89%)	42 (84%)	61 (86%)	23 (88%)
Present	13 (13%)	5 (11%)	8 (16%)	10 (14%)	3 (12%)
Lymph node metastasis, *n* (%)					
Absent	90 (93%)	43 (91%)	47 (94%)	64 (90%)	26 (100%)
Present	7 (7%)	4 (9%)	3 (6%)	7 (10%)	0
Washing cytology of pelvic cavity, *n* (%)					
Negative	84 (88%)	40 (87%)	44 (90%)	59 (86%)	25 (96%)
Positive	11 (12%)	6 (13%)	5 (10%)	10 (14%)	1 (4%)

*Note:* Two cases were missed in collecting washing cytology of pelvis cavity.

Abbreviations: BMI, body mass index; FIGO, International Federation of Gynecology and Obstetrics; MRI, magnetic resonance imaging; SLN, sentinel lymph node; SPECT, single photon emission computed tomography.

### 3.2 Univariate Analysis to Identify Factors Associated With SLN Detection Failure

A univariate logistic analysis was conducted to extract clinical factors associated with one‐sided or both‐sided SLN detection failure. No association was found in SLN detection failure with surgical modalities, period when surgery was performed, uterine tumor thickness measured using MRI, serum CA125, histological grade, or FIGO stage, myometrial invasion, lymphovascular invasion, and lymph node metastasis. However, age was significantly associated with SLN detection failure using the SPECT detection system, and age and BMI were significantly associated with SLN detection failure using the gamma probe detection system (Table 2). The SPECT detection system obtained an OR of 13.7 with a 95% CI of 4.61–40.7 for age (< 56 vs. ≥ 56) (*p* = 0.0001) (Table 2). Meanwhile, the gamma probe detection system obtained an OR of 8.62 with a 95% CI of 3.10–24.0 for age (< 56 vs. ≥ 56) (*p* = 0.0001) and an OR of 9.89 with a 95% CI of 2.72–36.0 for BMI (< 24.5 vs. ≥ 24.5) (*p* = 0.0005) (Table 2).

**TABLE 2:** Univariate and multivariate logistic regression analysis of predictors of SLN detection failure.

	SPECT		SPECT		Gamma probe		Gamma probe	
	Univariate analysis		Multivariate analysis		Univariate analysis		Multivariate analysis	
	OR (95% CI)	*p*	OR (95% CI)	*p*	OR (95% CI)	*p*	OR (95% CI)	*p*
Age								
< 56	Reference				Reference			
≥ 56	13.7 (4.61–40.7)	0.0001	12.9 (4.30–38.7)	0.0001	8.62 (3.10–24.0)	0.0001	7.89 (2.61–23.8)	0.0003
BMI								
< 24.5	Reference				Reference			
≥ 24.5	2.02 (0.90–4.56)	0.09	1.41 (0.54–3.65)	0.482	9.89 (2.72–36.0)	0.0005	8.97 (2.28–35.4)	0.0017
Surgical modality								
Laparoscopic	Reference				Reference			
Robotic‐assisted	0.74 (0.33–1.64)	0.197			1.12 (0.45–2.77)	0.808		
Period of surgery								
June 2019 to June 2021	Reference				Reference			
July 2021 to March 2023	1.13 (0.51–2.51)	0.763			0.97 (0.40–2.39)	0.951		
Thickness of tumor measured with MRI								
< 20	Reference				Reference			
≥ 20	1.40 (0.57–3.47)	0.465			2.15 (0.82–5.65)	0.121		
Serum CA125								
< 15	Reference				Reference			
≥ 15	0.68 (0.30–1.52)	0.345			0.51 (0.20–1.26)	0.143		
Histological grade								
1 and 2	Reference				Reference			
3 and clear cell carcinoma	NA	NA			NA	NA		
FIGO stage								
IA	Reference				Reference			
More than IB	0.93 (0.30–2.89)	0.900			1.11 (0.32–3.90)	0.872		
Myometrial invasion								
Absent	Reference				Reference			
Present	1.86 (0.82–4.22)	0.137			1.17 (0.47–2.94)	0.737		
Myometrial invasion depth								
< 1/2	Reference				Reference			
≥ 1/2	1.63 (0.37–7.23)	0.521			1.72 (0.38–7.78)	0.480		
Lymphovascular invasion								
Absent	Reference				Reference			
Present	1.60 (0.48–5.29)	0.441			0.80 (0.20–3.15)	0.745		
Lymph node metastasis								
Absent	Reference				Reference			
Present	0.69 (0.14–3.24)	0.635			NA	NA		
Washing cytology of pelvic cavity								
Negative	Reference				Reference			
Positive	0.76 (0.21–2.68)	0.666			0.24 (0.03–1.93)	0.180		

*Note: p* < 0.05 was considered statistically significant.

Abbreviations: BMI, body mass index; CI, confidence interval; FIGO, International Federation of Gynecology and Obstetrics; MRI, magnetic resonance imaging; NA, not applicable; OR, odds ratio; SLN, sentinel lymph node; SPECT, single photon emission computed tomography.

### 3.3 Multivariate Analysis to Determine Factors Associated With SLN Detection Failure

Multivariate logistic regression analysis was performed to determine clinical factors associated with one‐sided or both‐sided failure in SLN detection. Using a backward elimination method, age was identified to be strongly associated with SLN detection failure using the SPECT detection system, whereas age and BMI were found to be strongly associated with SLN detection failure using the gamma probe detection system (Table 2). The SPECT detection system obtained an OR of 12.9 with a 95% CI of 4.30–38.7 for age (< 56 vs. ≥ 56) (*p* = 0.0001) (Table 2). Meanwhile, the gamma probe detection system obtained an OR of 7.89 with a 95% CI of 2.61–23.8 for age (< 56 vs. ≥ 56) (*p* = 0.0003) and an OR of 8.97 with a 95% CI of 2.28–35.4 for BMI (< 24.5 vs. ≥ 24.5) (*p* = 0.0017) (Table 2).

### 3.4 Analysis of an SLN Detection Failure Cohort Using Preoperative SPECT

A specific cohort of SLN detection failure using a preoperative SPECT was analyzed for intraoperative SLN detection using a gamma probe. Among 50 cases, the intraoperative gamma probe failed to detect SLN unilaterally or on both sides in 23 cases (46%) (Table 3). Univariate logistic and multivariate logistic regression analyses were performed for the cohort. BMI was found to be strongly associated with SLN detection failure using the intraoperative gamma probe detection system, but not age. Univariate analysis showed an OR of 15.3 with a 95% CI of 2.96–78.8 (*p* = 0.0011), whereas multivariate analysis showed an OR of 14.7 with a 95% CI of 2.72–79.2 (*p* = 0.0018) for BMI (< 24.5 vs. ≥ 24.5) (Table 4).

**TABLE 3:** Demographic and clinical characteristics and intraoperative SLN detection of a cohort with unsuccessful SLN detection using SPECT.

		Gamma probe	Gamma probe
Group	Total	Detection success	Detection failure
Patients, *n*	50	27 (54%)	23 (46%)
Median age, years (range)	59.0 (32–80)	55.0 (36–80)	61.0 (32–80)
Median BMI (range)	26.3 (15.3–46.4)	23.8 (15.3–33.3)	28.7 (18.6–46.4)
Surgical modality, *n* (%)			
Laparoscopic	24 (48%)	14 (52%)	10 (43%)
Robotic‐assisted	26 (52%)	13 (48%)	13 (57%)
Number of surgeries each year, *n*			
2019	4	0	4
2020	13	8	5
2021	20	11	9
2022	12	8	4
2023	1	0	1
Thickness of tumor measured with MRI, mm (range)	12 (0–43)	9 (0–43)	14 (0–31)
Serum CA125, U/mL (range)	15.7 (5.5–103)	16.1 (5.5–103)	14.5 (8.2–66.8)
Histological grade, *n* (%)			
1 and 2	48 (96%)	25 (93%)	23 (100%)
3 and clear cell carcinoma	2 (4%)	2 (7%)	0
FIGO stage, *n* (%)			
IA	43 (86%)	23 (85%)	20 (87%)
More than IB	7 (14%)	4 (15%)	3 (13%)
IB	1 (2%)	0	2 (9%)
II	2 (4%)	1 (4%)	1 (4%)
IIIC1	2 (4%)	2 (7%)	0
IIIC2	1 (2%)	1 (4%)	0
Myometrial invasion, *n* (%)			
Absent	17 (34%)	9 (33%)	8 (35%)
Present	33 (66%)	18 (67%)	15 (65%)
Myometrial invasion depth, *n* (%)			
< 1/2	45 (91%)	24 (89%)	21 (91%)
≥ 1/2	5 (9%)	3 (11%)	2 (9%)
Lymphovascular invasion, *n* (%)			
Absent	42 (84%)	21 (78%)	21 (91%)
Present	8 (16%)	6 (22%)	2 (9%)
Lymph node metastasis, *n* (%)			
Absent	47 (94%)	24 (89%)	23 (100%)
Present	3 (6%)	3 (11%)	0
Washing cytology of pelvic cavity, *n* (%)			
Negative	44 (90%)	22 (85%)	22 (96%)
Positive	5 (10%)	4 (15%)	1 (4%)

*Note:* One case was missed in collecting washing cytology of pelvis cavity.

Abbreviations: BMI, body mass index; FIGO, International Federation of Gynecology and Obstetrics; MRI, magnetic resonance imaging; SLN, sentinel lymph node; SPECT, single photon emission computed tomography.

**TABLE 4:** Univariate and multivariate logistic regression analyses of predictors of intraoperative SLN detection failure of a cohort with unsuccessful SLN detection using SPECT.

	Gamma probe		Gamma probe	
	Univariate analysis		Multivariate analysis	
	OR (95% CI)	*p*	OR (95% CI)	*p*
Age				
< 56	Reference		Reference	
≥ 56	3.88 (1.12–13.5)	0.0330	3.62 (0.87–15.1)	0.0777
BMI				
< 24.5	Reference		Reference	
≥ 24.5	15.3 (2.96–78.8)	0.0011	14.7 (2.72–79.2)	0.0018
Surgical modality				
Laparoscopic	Reference			
Robotic‐assisted	1.40 (0.46–4.28)	0.555		
Period of surgery				
June 2019 to June 2021	Reference			
July 2021 to March 2023	0.85 (0.28–2.59)	0.777		
Thickness of tumor measured with MRI				
< 20	Reference			
≥ 20	1.52 (0.45–5.14)	0.497		
Serum CA125				
< 15	Reference			
≥ 15	0.62 (0.20–1.89)	0.396		
Histological grade				
1 and 2	Reference			
3 and clear cell carcinoma	NA	NA		
FIGO stage				
IA	Reference			
More than IB	0.86 (0.17–4.33)	0.857		
Myometrial invasion				
Absent	Reference			
Present	0.94 (0.29–3.03)	0.914		
Myometrial invasion depth				
< 1/2	Reference			
≥ 1/2	0.76 (0.12–5.01)	0.777		
Lymphovascular invasion				
Absent	Reference			
Present	0.33 (0.06–1.84)	0.208		
Lymph node metastasis				
Absent	Reference			
Present	NA	NA		
Washing cytology of pelvic cavity				
Negative	Reference			
Positive	0.25 (0.03–2.42)	0.231		

*Note: p* < 0.05 was considered statistically significant.

Abbreviations: BMI, body mass index; CI, confidence interval; FIGO, International Federation of Gynecology and Obstetrics; MRI, magnetic resonance imaging; NA, not applicable; OR, odds ratio; SLN, sentinel lymph node; SPECT, single photon emission computed tomography.

### 3.5 SLN Detection Sites

**FIGURE 1:**
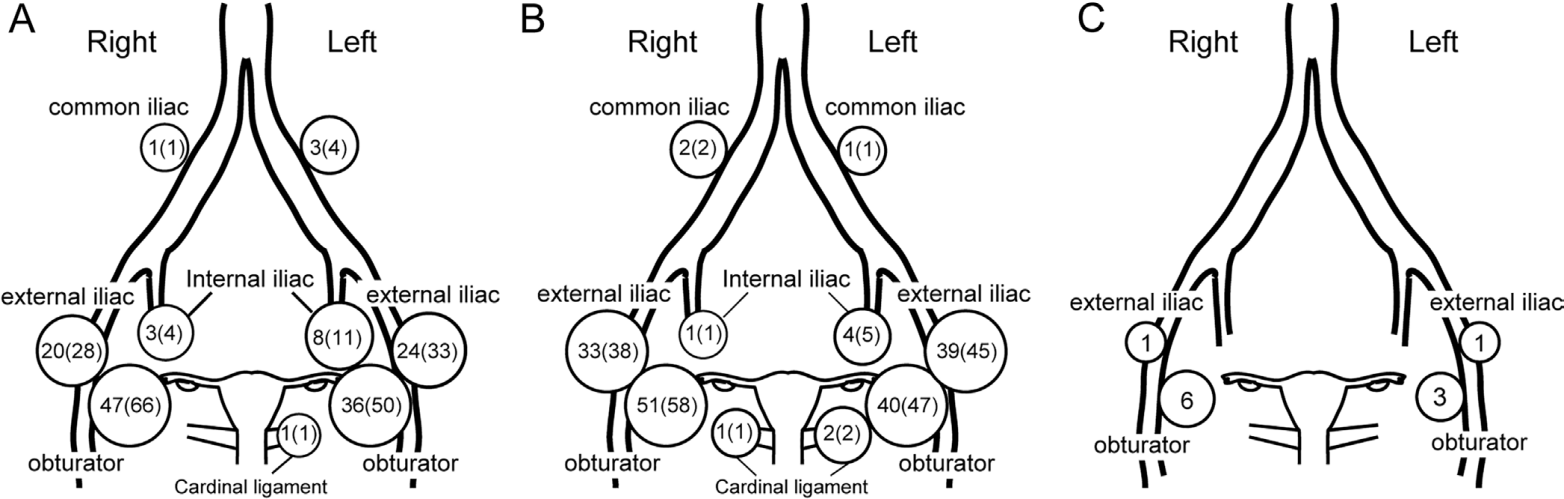
Locations of detected SLN and metastasis‐positive lymph nodes. (A, B) Schematic presentation of location and number of intraoperatively detected SLN using the preoperative SPECT (A) and intraoperative gamma probe (B). Numbers in brackets indicate percent values of the total SLN in the pelvic hemisphere. (C) The location and number of pathologically diagnosed metastasis‐positive lymph nodes.

Out of 97 patients, a preoperative SPECT detected SLN in 88 on either or both sides. Including cases with multiple SLN sites in a pelvic hemisphere, 11 SLNs were detected in the internal iliac area, 44 in the external iliac area, 83 in the obturator area, 1 in the cardinal ligament area, 4 in the common iliac, and none in the paraaortic areas (Figure 1A). Furthermore, out of 97 patients, 94 finally underwent surgery with detectable SLN on either or both sides using a gamma probe. Including cases with multiple SLN sites in a pelvic hemisphere, 5 SLNs were detected in the internal iliac area, 72 in the external iliac area, 91 in the obturator area, and 3 in the cardinal ligament area, 3 in the common iliac area, and none in the paraaortic area (Figure 1B). Seven cases were positive for lymph node metastasis. Among the seven cases, four were positive for lymph node metastasis on both sides. Three cases were diagnosed positive for lymph node metastasis intraoperatively and converted into open surgery. The remaining four cases were diagnosed positive for lymph node metastasis postoperatively, and adjuvant therapy was applied. Among these cases, two were micrometastasis which appeared by postoperative reexamination, whereas the remaining two could not be differentiated from endosalpingiosis intraoperatively. Immunohistochemical analyses using several antibodies against PAX8, estrogen receptor, progesterone receptor, p53, and WT1 were used to compare with original lesions for the final diagnosis. Figure 1C illustrates the sites and number of metastasis‐positive SLN. All the metastasis‐positive SLNs were located either in the external iliac or obturator area (Figure 1C).

## 4 Discussion

This study investigated the association between SLN detection failure and various clinical factors of early‐staged endometrial cancer cases who underwent MIS at our institute. As a result, we extracted critical clinical factors, including age and BMI, for predicting SLN detection failure. Several studies reported that BMI [12, 13, 14, 15], age [12, 16], nonendometrioid histology [12, 13, 17], lymphovascular invasion [12, 17], and lymph node metastasis [13] are associated with SLN detection failure. Postoperative diagnosis sometimes differed from preoperative diagnosis as Stage IA/pathological Grade 1 or 2, but our cohort was rather uniform in terms of clinical stage and pathological grading. Hence, ratios of non‐endometrial histology, lymphovascular invasion, and lymph node metastasis were very small. Under this limited condition, age and BMI were considered remarkable clinical factors associated with SLN detection failure. We analyzed the data from the SPECT imaging conducted 1 day before surgery and the intraoperative gamma probe detection. Although strong associations between SLN detection failure and age and BMI were observed in the intraoperative gamma probe data, no remarkable association between BMI and SLN detection failure was found in the preoperative SPECT data. However, when limited to the preoperative SLN detection failure cohort, there was a strong association between intraoperative SLN detection failure and BMI, but not age. These results suggest that age determined early and lasting SLN detection, whereas BMI determined only late SLN detection. Based on our results, obesity but not age may slow lymphatic flow in the pelvis. This is the first study reporting detailed detection sites of the two modalities, the SPECT and gamma probe, and clinical factors associated with SLN detection failure for each modality.

The SLN detection rates in this study were comparable to other studies using ^99m^Tc colloid injections into the uterine cervix for estimated early‐stage endometrial cancer cases (Table 5) [16, 19, 22, 23]. Two of them studied clinical factors associated with SLN detection failure. Martinez et al. found age to be associated with SLN detection failure, but not BMI (Table 5) [16]. Sahbai et al. found bone marrow uptake of radioisotope to be associated with SLN detection failure, but not age and BMI (Table 5) [19]. These variations in data may be caused by differences in the characteristics of each cohort.

**TABLE 5:** SLN detection rates and clinical factors associated with SLN detection failure.

		SPECT	SPECT	Gamma probe	Gamma probe	Factors associated with SLN detection failure
Authors	*n*	Overall detection	Bilateral detection	Overall detection	Bilateral detection	
Sahbai^a^	145	76%	NA	74%	NA	Bone marrow uptake
						Peritoneal/abdominal radioactivity
Martinez^b^	92	86%	40%	NA	NA	Age, myometrial invasion
Togami^c^	122	NA	NA	96%	88%	NA
Frati^d^, ^e^	118	75%	37%	86%	53%	NA
This study	97	91%	48%	97%	73%	Age, BMI

Abbreviations: BMI, body mass index; NA, not applicable; SPECT, single photon emission computed tomography.

^a^ Sahbai et al. [19].

^b^ Martínez Bravo et al. [16].

^c^ Togami et al. [22].

^d^ Frati et al. used ^99m^Tc colloid plus patent blue.

^e^ Frati et al. [23].

The underlying pathophysiological mechanism remains unclear, but SLN detection failure in aged patients and those with obesity may be associated with lymphatic dysfunction [15, 24]. Age and obesity were strongly associated with lymphatic dysfunction [25, 26, 27]. Empty packet dissection, which is the absence of lymph nodes on the final pathological diagnosis of the tissue sample submitted as SLN, was positively associated with BMI [15]. Empty packet dissection may cause SLN detection failure associated with BMI. In our study, we promptly removed fat tissue from the lymph nodes and confirmed the presence of lymph nodes before submitting them for intraoperative pathological examination. Therefore, we did not encounter any cases with empty packets. Cabrera et al. reported that using ^99m^Tc colloid reduced the incidence of empty packets to 1.9% [28]. Theoretically, as are relatively large particles, radiotracers remain still in SLNs and do not extravasate.

Our analysis revealed that > 90% of detected SLN were located in the external iliac area or obturator area, which was consistent with previous studies using cervical injection of tracer [29, 30, 31]. All metastasis‐positive lymph nodes in our study were located either in the external iliac or obturator area. The above studies included a small number of cases, in which paraaortic lymph nodes were detected as SLN [29, 30, 31]. In contrast, our study did not detect any paraaortic lymph node as SLN. This discrepancy may be due to the difference in injection technique. The NCCN guideline recommended a combined cervical injection of superficial (1–3 mm) and deep (1–2 cm) sites to deliver the tracer to the main lymphatic channel layer in the cervix and uterus [8, 29]. Cervical injection significantly increased the SLN detection rate compared to injection into the corpus [32]. We would like to emphasize that our technique is mainly applicable to low‐risk endometrial cancer cases, which have an extremely low risk of paraaortic lymph node metastasis [33, 34, 35].

Based on our study, we extract patient groups with a high risk of SLN mapping failure. Some technical modifications related to tracer administration could be considered for such cases. Combined use of multiple tracers would increase the detection success rate [36]. Repeated injection of the same or different tracer may be considered if SLN is not detected after the first injection. Corpus injection may be considered if SLN is not detected after cervical injection [16].

Our study has several limitations. First, the study was based on a limited size of cohort from an institute. The cohort was limited to preoperatively estimated low‐risk Stage IA/pathological Grade 1 or 2 cases. Age and BMI may be extracted by multivariate regression analysis because of this uniform cohort regarding disease aggressiveness. Moreover, we only used a subepithelial ^99m^Tc colloid injection into the uterine cervix. We did not analyze the long‐term prognosis of the cohort because of the short follow‐up period. We also did not perform backup lymphadenectomy for all the cases.

In conclusion, the SLN biopsy system was effectively applied to patients with endometrial cancer. Attempts to improve SLN identification in elderly patients and those with obesity are warranted to obtain maximum benefits of MIS for low‐ or medium‐risk cases.

## Author Contributions

All authors agree with the content of the manuscripts. Concept and design of study and acquisition of data and analysis and interpretation of data; drafting the article and revising it critically: K.A. and H.Y. Acquisition of data and revising the article critically for important intellectual content: K. Kodama, K.O., M.Y., I.O., H.Y., S.M., and K.H. Statistical analysis and interpretation of data: M.S. Final approval of the version to be published: K.I., Y.O., and K. Kato.

## Conflicts of Interest

The authors declare no conflicts of interest.

## Data Availability

The data that support the findings of this study are available from the corresponding author upon reasonable request.
